# A Deep Learning Approach to Measure Visual Function in Zebrafish

**DOI:** 10.3390/biology14060663

**Published:** 2025-06-09

**Authors:** Manjiri Patil, Annabel Birchall, Hammad Syed, Vanessa Rodwell, Ha-Jun Yoon, William H. J. Norton, Mervyn G. Thomas

**Affiliations:** 1 The University of Leicester Ulverscroft Eye Unit, School of Psychology and Vision Sciences, University of Leicester, Robert Kilpatrick Clinical Sciences Building, P.O. Box 65, Leicester LE2 7LX, UK; 2 Neurogenetics Group, Department of Genetics, Genomics and Cancer Sciences, University of Leicester, Robert Kilpatrick Clinical Sciences Building, P.O. Box 65, Leicester LE2 7LX, UK

**Keywords:** zebrafish, optokinetic response, visual behaviour, visual development, deep learning

## Abstract

Visual behaviour in zebrafish, often measured by the optokinetic reflex (OKR), serves as a valuable model for studying aspects of human neurological and ocular diseases and for conducting therapeutic or toxicology assays. Traditional methods for OKR analysis often rely on binarization techniques (threshold-based conversion of images to black and white) or costly software, which limits their utility in low-contrast settings or hypopigmented disease models. Here, we present a novel deep learning pipeline for OKR analysis, using ResNet-50 within the DeepLabCut framework in a Python Version 3.10 environment. Our approach employs object tracking to enable robust eye movement quantification, regardless of variations in contrast or pigmentation. OKR responses were elicited in both wild-type and *slc45a2* (albino) mutant zebrafish larvae at 5 days post-fertilisation, using a mini-LED arena with a rotating visual stimulus. Eye movements were recorded and analysed using both conventional software and our deep learning approach. We demonstrate that the deep learning model achieves comparable accuracy to traditional methods, with the added benefits of applicability in diverse lighting conditions and in hypopigmented larvae. Statistical analyses, including Bland–Altman tests, confirmed the reliability of the deep learning model. While this study focuses on 5-day-old zebrafish larvae under controlled conditions, the pipeline is adaptable across developmental stages, pigmentation types, and behavioural assays. With appropriate adjustments to experimental parameters, it could be applied to broader behavioural studies, including social interactions and predator–prey dynamics in ocular and neurological disease models.

## Simple Summary

The optokinetic reflex (OKR) assay is a valuable tool for investigating aspects of human neurological and ocular diseases. Traditional OKR analysis pipelines are reliant on contrast-based binarization or expensive software and often struggle in low-contrast environments. We utilised DeepLabCut (DLC v2.3.9), a robust object tracking model, in the OKR pipeline for accurately quantifying larval eye movements in zebrafish. We demonstrate comparable accuracy of the DLC model to other traditional methods with superior reliability, especially in diverse lighting, in wild-type as well as hypopigmented mutant zebrafish. The adaptability of this pipeline suggests its utility beyond visual behaviours, such as in social interactions and predator–prey dynamics, further benefiting research on ocular and neurological disease models.

## 1. Introduction

The optokinetic reflex (OKR) is a compensatory eye movement that occurs in response to a large-field moving visual stimulus [1]. This reflex stabilises images on the retina, thereby maximising visual acuity, and is triggered by a drifting visual scene that is detected by retinal ganglion cells. During OKR, the eyes move slowly in the direction of the stimulus followed by a fast corrective saccade in the opposite direction, forming a negative feedback loop. The OKR works in conjunction with the vestibulo-ocular reflex to maintain a stable line of sight during head and body movements [2].

OKR assays, which use full field moving visual stimuli to elicit these reflexes, are widely employed to assess visual acuity, oculomotor control and sensorimotor integration. OKR is an important indicator of visual function and is commonly used in research to study visual processing and related disorders [3,4,5]. Zebrafish (*Danio rerio*) have emerged as a powerful model for visual neuroscience and disease modelling, owing to their optical transparency, external development and high genetic homology with humans. Approximately 71% of human protein-coding genes have at least one zebrafish orthologue, and 82% of human disease-associated genes listed in the Online Mendelian Inheritance in Man (OMIM) database are represented in the zebrafish genome [6]. Zebrafish exhibit rapid development, high fecundity, and low maintenance costs, facilitating genetic manipulation and large-scale studies [7]. Their ocular anatomy and physiology share similarities with humans, making them valuable for modelling neurological and ocular diseases [8]. Notably, the optokinetic response (OKR) can be measured as early as 3 days post-fertilisation (dpf), with robust responses typically observed by 5 dpf [9].

Traditional methods for analysing larval eye movements involve manual annotation or semi-automated software, both of which present limitations. Manual analysis is labour-intensive and subjective, while existing automated tools rely heavily on contrast-based image binarization. Two widely used open-source tools, OKRtrack (MATLAB-based v2019a) [10] and Stytra (Python-based) [11], binarise the image assuming that larval eyes appear significantly darker than the background. While Stytra offers adjustable contrast thresholds and ellipse fitting to calculate eye angles, both tools are susceptible to inaccuracies in low-contrast scenarios or in hypopigmented models. Additionally, MATLAB requires a commercial licence, potentially limiting accessibility.

These limitations affect reproducibility and generalisability, particularly when imaging conditions or pigmentation vary—as is common in disease models such as slc45a2 mutants or albinism analogues. To address these challenges, we developed a deep learning-based analysis pipeline leveraging ResNet-50 within the DeepLabCut framework [12]. This approach uses object tracking, where specific anatomical landmarks are followed across frames based on their learned appearance, independent of image contrast, enabling accurate and unbiased tracking of larval eye movements across a range of pigmentation levels and lighting conditions. Here, we compare the performance of our deep learning pipeline with traditional methods in both wild-type and hypopigmented slc45a2 mutant zebrafish larvae.

## 2. Materials and Methods

### 2.1. Animal Ethics and Husbandry

All procedures adhered to the Animals in Scientific Procedures Act 1986 and were conducted under project licence PP1567795 by researchers holding individual UK Home Office personal licences.

Adult zebrafish (*Danio rerio*) were maintained in the Preclinical Research facility at the University of Leicester under standard conditions. Breeding was initiated by separating adult males and females overnight with a divider. Fertilised eggs were collected the following morning after removal of the divider. Embryos were maintained in 10-cm Petri dishes containing fish water (0.3 g/L Instant Ocean) at 28.5 °C until 5 days post-fertilisation (dpf).

For CRISPR knockout experiments, wild-type AB strain zebrafish were used. Embryos were injected at the single-cell stage with gRNA/Cas9 ribonucleoprotein (RNP) complexes to generate *slc45a2* knockouts, which exhibit hypopigmentation. A total of 25 confirmed *slc45a2* knockout embryos were used for OKR experiments.

### 2.2. Generation of slc45a2 CRISPR F0 Knockout Larvae

CRISPR-based knockout of *slc45a2* was performed following the protocol described by François Kroll et al. 2021 [13]. Three synthetic crRNAs targeting distinct regions of exons of *slc45a2* were selected from the Integrated DNA Technologies (IDT) (Coralville, IA, USA) predesigned database based on high on-target scores, low off-target potential, and relevance to functional domains (Supplementary Table S1). RNP complexes were prepared by combining equimolar amounts of the three crRNAs with GFP-tagged Cas9 protein. Approximately 1 nL of the mixture was injected into single-cell stage embryos using borosilicate glass capillaries (1.0 mm OD × 0.78 mm ID × 100 mm L) and an μPUMP microinjector (PN99322, World Precision Instruments (Sarasota, FL, USA)). GFP-tagged Cas9 protein was used to confirm successful uptake using a NIGHTSEA royal blue light source and filter approximately 4 h post-injection. *slc45a2* knockout was confirmed based on hypopigmentation phenotype observed via microscopy.

### 2.3. Optokinetic Reflex (OKR) Assay

At 5 dpf, zebrafish larvae were carefully placed with their dorsal side up in 4–5% methylcellulose within a 35 mm Petri dish to immobilise the body while allowing unrestricted eye movement. Immobilised larval zebrafish were placed on the stage of a Leica S9i digital stereo microscope (Wetzlar, Germany) for video capture.

Visual stimuli were presented using three mini-LED screens (dimensions: W80 mm × H50 mm) arranged at right angles to form a partial arena. Vertical black-and-white sinusoidal gratings (Supplementary Tables S2 and S3) were generated using PsychoPy [14] (v2023.1.0). Videos were recorded using the LeicaS9i microscope. Lights from a TL3000 Ergo base (Leica Microsystem (Wetzlar, Germany)) were used for standard visible-spectrum brightfield illumination, while a modified set-up (Section 2.6) was developed to record eye movements under non-visible light conditions. The larva was placed in front of the stimulus, ensuring both eyes were in the recoding field of the camera.

### 2.4. DeepLabCut Training for Eye Movement Tracking

Video recordings were analysed using DeepLabCut [12] (DLC v2.3.9; https://github.com/DeepLabCut/DeepLabCut accessed on 7 June 2024), a deep learning-based markerless tracking toolkit built on ResNet 50. A dataset of 200 annotated frames comprising both wild-type (pigmented) and *slc45a2* mutant (hypopigmented) larvae was used to train the network. Key points were annotated for both eyes (LE1, LE2, RE1, RE2) and mid-body (MID1, MID2). Training was conducted over 200,000 iterations. Since the DLC software (v2.3.9) performs an integrated training–validation split, no manual data split was applied. The model’s performance was evaluated using the built-in test error on unseen frames, and training convergence was monitored throughout the 200,000 iterations.

Conventional cross-validation strategies such as k-fold or leave-one-out were not used, as they are not standard practice for pose estimation models trained on video data. Video frames are often temporally correlated, and dividing them into artificial folds does not provide meaningful additional validation. DeepLabCut’s internal split is optimised for evaluating generalisation across varied image contexts.

To enhance model efficiency, frames were augmented with randomised zoom levels, contrast adjustments, and image transformations (rotation, cropping, embossing, scaling). The learning rate was initially set at 0.005 for 10,000 iterations and gradually increased to 0.02.

We conducted all analyses on a workstation equipped with an Intel Core i7-9800X processor, 32 GB RAM, and an NVIDIA Quadro P4000 GPU (Intel Corporation, Santa Clara, CA, USA). This setup provided efficient offline processing speeds of 12–15 FPS for high-resolution video, enabling rapid analysis while maintaining precision in slow-phase velocity extraction. While real-time pose estimation was not required for our offline OKR assay analysis, DeepLabCut also supports live tracking applications through its companion package DLC-Live (https://github.com/DeepLabCut/DeepLabCut-live accessed on 19 May 2025), which may be useful for other experimental contexts.

### 2.5. Comparative Analysis Using Traditional Software and DeepLabCut

All OKR recordings were processed using three methods: (i) OKRtrack (MATLAB-based), (ii) Stytra (Python-based), and (iii) our trained DeepLabCut model. For DLC analysis, a zero-phase low-pass filter (10 Hz cut-off) was applied to the rotational time series data, followed by boxcar smoothing. Numerical differentiation was then performed on the smoothed signal to derive eye rotation velocity. Slow and fast phase eye movements were identified using the second-order derivate peak detection algorithm. For each light condition, eye movement velocity was quantified by performing linear regression between peak and trough on the smoothed angular data. These analyses were conducted using custom Python scripts (v3.9) and visualised with Matplotlib (v3.7.1). For MATLAB and Stytra v0.8, the inbuilt processing algorithms were used. For comparison between DLC v2.3.9 and OKRtrack, slow-phase velocities during the 60 s clockwise stimulus (Supplementary Table S3) were calculated. The agreement between methods was assessed using Bland–Altman analysis. Statistical analyses were performed in RStudio (v2024.04.2+764).

### 2.6. Modified OKR Assay Using Infrared Illumination

To test model performance under non-visible light conditions, a modified OKR assay was developed using infrared (IR) backlighting. An 850 nm IR emitter (PHLOX^®^ LEDIR850-BL-50X50-LLUB-QR-24V (Aix-en-Provence, France); dimensions: 50 mm × 50 mm) was placed beneath the Petri dish to minimise ambient visual noise. All assays were conducted in the dark between 1:00 and 4:00 p.m. to standardise environmental lighting.

### 2.7. Comparison of Eye Movements Under Visible vs. IR Illumination

To compare the performance of eye tracking under visible versus IR conditions, larvae were recorded under both lighting setups and analysed via the DeepLabCut pipeline. Eye movement coordinates were extracted and used to calculate slow-phase eye velocities using a custom Python script. Data distributions were assessed for normality, followed by Mann–Whitney U tests to evaluate statistical significance (*p* < 0.05) between groups.

For consistency, all reported values represent left eye slow-phase velocity, as results were comparable between eyes. Data are presented as mean ± standard error of the mean (SEM).

## 3. Results

### 3.1. Training and Evaluation of the DeepLabCut (DLC) Model

**Figure 1:**
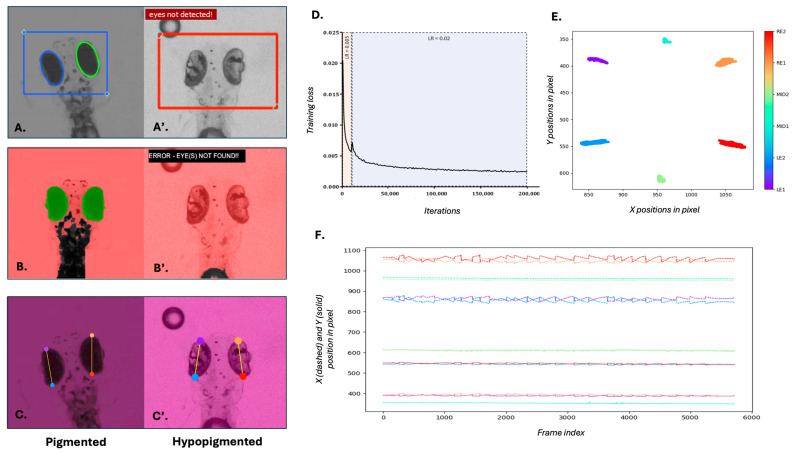
**Comparison of eye tracking performance and model training using traditional and deep learning methods.** (**A**–**C**) Eye detection and tracking in wild-type 5 dpf zebrafish larvae using (**A**) Stytra, (**B**) OKRtrack and (**C**) DeepLabCut (DLC). (**A’**–**C**’) Eye detection and tracking in slc45a2 hypopigmented larvae using the same three methods. (**A’**,**B’**) failed to detect the eye in hypopigmented larvae, as indicated by error messages and absence of overlays. In contrast, DLC (**C’**) successfully identified key anatomical landmarks across pigmentation levels. (**D**) Training loss curve over 200,000 iterations during DLC model training. The black line represents the training loss; shaded areas represent the two different learning rates. The plot shows a steep initial decline in the training loss, indicating rapid learning. As training progresses, the rate of decline slows down and the training loss gradually converges, suggesting that the model is approaching its optimal performance. (**E**) Cluster plot showing relative positions of tracked landmarks across the visual stimulus duration. Colours correspond to specific key points annotated for left eye (LE1, LE2), right eye (RE1, RE2) and mid-body (MID1, MID2) as indicated in the colour bar. Eye landmarks show high positional changes to the visual stimulus, while body landmarks remain stable. (**F**) Time-series plot of X (dashed lines) and Y (solid lines) positions of all six landmarks across frames. Midline body points remain relatively stable, while eye landmarks exhibit characteristic OKR waveforms in response to the visual stimulus.

We trained a novel deep learning model using DeepLabCut (v2.3.9), based on ResNet-50 architecture, to track anatomical landmarks on both pigmented and hypopigmented 5 dpf zebrafish larvae. Specifically, the model aimed to locate two points on the left eye (LE1, LE2) and two points on the right eye (RE1, RE2) (Figure 1C,C’) over 200,000 training iterations. To enhance the model’s robustness, the training dataset incorporated images with varying zoom levels and contrast. A staged learning rate strategy was used, beginning at 0.005 for the initial 10,000 iterations and increasing to 0.02 to optimise learning. The training loss decreased consistently over 200,000 iterations, demonstrating effective convergence (Figure 1D). Moreover, the close clustering of predicted landmark positions across frames (Figure 1E) indicates the model’s high precision and consistency in anatomical localisation.

This trained DLC model successfully tracked eye positions across time, generating precise spatiotemporal plots of movement. The dynamic changes in larval eye movements over time were observed with a characteristic “sawtooth” appearance of the OKR quick and slow phases (Figure 1F).

### 3.2. Comparison with Traditional Eye Tracking Methods: Stytra and OKRtrack

**Figure 2:**
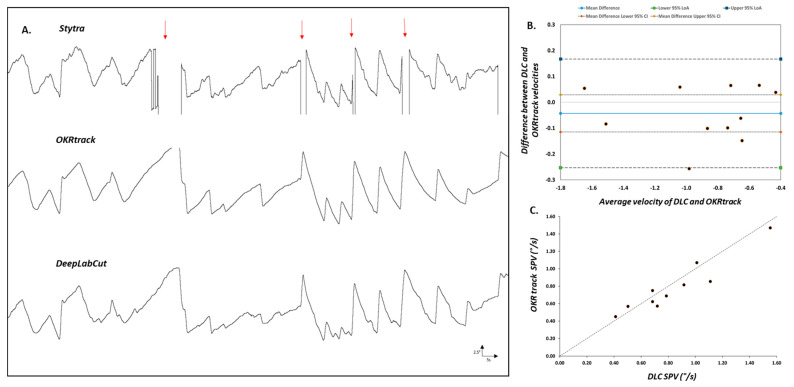
**Comparison of eye tracking using traditional software and the DeepLabCut model.** (**A**) Representative eye movement traces of larval zebrafish recorded during the optokinetic reflex (OKR) assay and analysed using three different pipelines: Stytra (top), OKRtrack in MATLAB (middle) and DeepLabCut (bottom). Stytra traces show missing segments (highlighted by red arrows), likely due to its reliance on image contrast thresholds and elliptical shape fitting. In contrast, both OKRtrack and DLC produce smoother and more complete traces of eye position over time, with consistent tracking of the slow-phase and saccadic components of the OKR. (**B**) Bland–Altman agreement plot comparing OKRtrack and DLC models. Each point represents the difference in slow-phase eye velocity between the two methods plotted against the mean value. The solid blue line denotes the mean difference (−0.043), while the outer dashed lines represent the 95% limits of agreement (−0.25 to 0.16), indicating good concordance between the methods. (**C**) Scatter plot comparing absolute slow-phase eye velocity values calculated by DeepLabCut (DLC) and OKRtrack for each fish. The diagonal line represents the line of identity (y = x), indicating perfect agreement. The results support the interchangeable use of DLC and OKRtrack for eye movement quantification in zebrafish OKR assays.

To benchmark the DLC pipeline, we compared its performance with two established tools, Stytra and OKRtrack, using OKR recordings from both wild-type (WT) and *slc45a2* hypopigmented zebrafish larvae. The direct comparison revealed differences in their data processing abilities. Stytra exhibited missing data when analysing pigmented WT larvae (Figure 2A), suggesting its potential limitations compared to OKRtrack and the DLC model.

#### 3.2.1. Bland–Altman Analysis

The agreement between OKRtrack and the DLC model was assessed using a Bland–Altman plot, a method commonly used in clinical research to quantify agreement between two measurement techniques. Data points were evenly distributed around the mean difference (solid blue line), with limits of agreement ranging from −0.25 to 0.16, indicating strong concordance and suggesting that both methods yield comparable estimates of eye movement velocity (Figure 2B).

#### 3.2.2. Hypopigmented Fish

In hypopigmented *slc45a2* mutants, both Stytra and OKRtrack failed to consistently detect the eyes, likely due to their dependence on contrast-based segmentation, i.e., separating bright and dark regions based on pixel intensity and ellipse fitting. In contrast, the DLC model successfully tracked eye movements in all cases, highlighting its adaptability across varying pigmentation levels and experimental lighting conditions.

### 3.3. Infrared Backlighting Enhances OKR Behavioural Response Robustness

**Figure 3:**
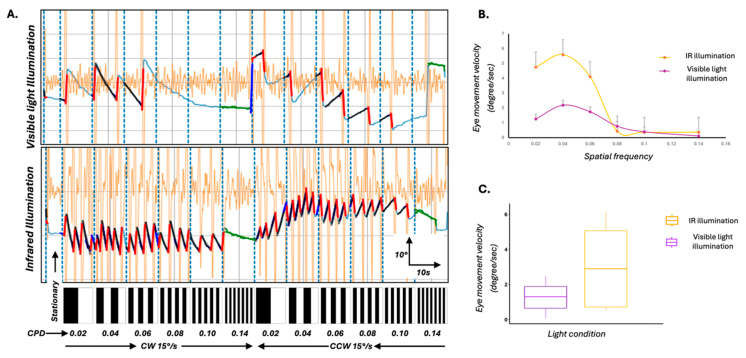
**Optimisation of the OKR assay using infrared (IR) illumination to enhance behavioural response robustness.** (**A**) Representative eye movement traces of 5 dpf wild-type zebrafish larvae exposed to full-field moving sinusoidal gratings under two lighting conditions: standard visible light (top) and IR illumination (bottom). Vertical blue dashed lines mark transitions in spatial frequency and direction of stimulus motion. Larvae under IR illumination exhibit clearer, higher slow-phase velocity optokinetic responses across stimulus epochs. Black overlays on the waveform represent the average slow phases and the red line represents the quick phases. Underlying eye movement trace is represented by light blue line. Orange waveforms represent the velocity trace. The bar below each panel illustrates the temporal sequence of stationary and moving gratings, with increasing spatial frequency (in cycles/degree (CPD) from left to right) and directional changes (clockwise (CW) and counterclockwise (CCW)). (**B**) Mean slow-phase eye velocity of the left eye as a function of spatial frequency under IR (yellow) and visible light (purple) conditions. IR illumination led to significantly enhanced eye velocities, particularly at intermediate spatial frequencies. Error bars represent standard error of the mean. (**C**) Box plots comparing overall eye movement velocity under IR and visible light conditions. Larvae tested under IR illumination showed significantly higher tracking velocities (*p* < 0.05), supporting the effectiveness of IR backlighting in enhancing OKR behavioural responses by reducing background noise and improving stimulus contrast.

To investigate whether changes to the experimental lighting setup could enhance the robustness of the OKR response itself, we modified the standard assay by introducing an 850 nm infrared (IR) backlight positioned beneath the zebrafish. This illumination approach allowed clear imaging of the larvae without interfering with the visual stimulus (the only visible light source in the arena) and also minimised the photoreceptor bleaching that can occur under bright ambient lighting. We compared OKR responses under two lighting conditions—standard visible light and IR backlight—in eight 5 dpf larvae. Under IR, larvae demonstrated more robust optokinetic behaviour, characterised by clearer eye movement traces and significantly increased slow-phase velocities (Figure 3A–C; *p* < 0.05, Mann–Whitney U test).

These results suggest that IR illumination enhances the biological response to visual stimuli, and may improve stimulus contrast and reduce background visual noise.

## 4. Discussion

The OKR assay, which uses a full-field moving visual stimulus to elicit a visual reflex, is a valuable tool to assess visual acuity, oculomotor control and behaviour in animals. In this study, we successfully developed a novel deep learning pipeline using DeepLabCut to analyse zebrafish larval eye movements, offering a cost-effective and precise alternative to traditional methods. The novel DLC model displays enhanced reliability compared to Stytra and comparable accuracy to OKRtrack when tracking eye movements in wild-type zebrafish. In agreement with this, Bland–Altman analysis confirmed the agreement between DLC and OKRtrack, supporting DLC’s reliability as a tool for eye movement analysis. The training process employed extensive data augmentation (including variations in contrast, zoom, and orientation) and showed steady loss convergence across 200,000 iterations, with no indication of overfitting. Additionally, the overall 95% limits of agreement (−0.26 to 0.16) remained tight around the mean difference (−0.043), indicating no systematic bias. Furthermore, the data points were distributed fairly evenly around the mean difference, suggesting no bias across the range of measurements. Although our pipeline performed robustly across all standardised recordings, we observed occasional tracking errors when deviations from protocol occurred. These included cases where the eyes were not fully visible in the recording frame, multiple larvae were accidentally present, or the methylcellulose concentration was too low, leading to excessive body movement or bubble artefacts that mimicked eye landmarks. These events were rare (<2%) and were resolved by ensuring proper setup and controlled assay conditions. We note these not as limitations of the model itself, but as reminders that even deep learning pipelines benefit from high-quality input data and strict adherence to experimental best practices. DLC also showed a distinct advantage in accurately tracking hypopigmented mutant larvae, where traditional methods failed due to their reliance on image contrast and eye shape. Finally, the incorporation of an infrared light source in the OKR assay setup proved effective in minimising background noise, enhancing the efficiency and robustness of the assay.

These findings align with a broader trend in small model organism research: the integration of deep learning and computer vision to improve behavioural tracking. Recent applications in zebrafish, *Drosophila*, and *C. elegans* have shown that neural networks like ResNet, YOLO, DANNCE and DeepPoseKit can achieve markerless pose estimation, even in challenging imaging environments [12,15,16,17,18,19]. A comparative summary of visual tracking methods are shown in Supplementary Table S4. DeepLabCut, in particular, has become a popular tool for behavioural quantification across species, offering user-defined key point tracking and high flexibility. Its adaptability to varying conditions (pigmentation levels, zoom, and lighting), sub-pixel accuracy, and the ability to leverage pre-trained networks to reduce training data, outweighing the need for manual data annotation—demonstrated in this study—makes it especially suited for reproducible phenotyping in genetic models, including assays of locomotion, social interaction, and predator–prey behaviour.

Commercial platforms such as Noldus EthoVision XT (Amersfoort, The Netherland) and ViewPoint ZebraLab (Civrieux, France) offer useful tools for behavioural analysis, but they often require proprietary hardware, incur high costs, and offer limited adaptability for custom assays [17]. In contrast, our pipeline is cost-effective, open-source, and adaptable to a wide range of experimental setups, providing a democratised solution for laboratories lacking access to expensive commercial tools. The recent emergence of compact OKR systems using LED displays or smartphone-derived screens supports this move toward portable, flexible, and affordable behavioural assays [20].

While our deep learning model was trained with extensive data augmentation to simulate variation in imaging conditions, the OKR assay itself was deliberately conducted under tightly controlled lighting and environmental parameters. This is in line with standard practice for OKR studies, where uncontrolled variables such as ambient light, water turbidity, or background motion can compromise reflex elicitation and reproducibility. Our goal was to optimise the robustness of behavioural readouts in biologically relevant and standardised experimental settings, rather than introduce artificial variability.

Beyond technical improvements, enhanced OKR tracking offers direct benefits for biomedical research. Zebrafish are widely used to model human eye diseases—including inherited retinal disorders and optic nerve pathologies—as well as neurological conditions like epilepsy, autism, and neurodegeneration [4,21,22]. Accurate quantification of eye movements enables the detection of subtle phenotypes, such as nystagmus-like phenotypes [23] and impaired slow-phase velocity, which can indicate specific functional impairments in visual or brain circuits. Improved behavioural resolution also enhances zebrafish’s value in phenotypic drug screening [24]. High-throughput platforms using larval zebrafish have already leveraged deep learning to identify pharmacological agents that reverse disease-associated behaviours or affect neurotransmission pathways [15,16,25].

Our pipeline represents a notable advance over traditional OKR analysis methods such as Stytra and OKRtrack due to its pigment-agnostic tracking capability. This feature is particularly valuable in zebrafish research, where chemical depigmentation (e.g., via PTU) is commonly used to enhance anatomical visibility in the eyes and brain. Unlike contrast-dependent systems, our DeepLabCut-based approach reliably tracks eye movements in hypopigmented larvae, enabling accurate phenotyping even in low-contrast conditions. Given the known association between hypopigmentation and various ocular diseases, our assay could be applied to screen for compounds that restore visual behaviour in mutant larvae or identify toxic effects on the visual system. Furthermore, automated pipelines allow large-scale data generation suitable for unsupervised behavioural fingerprinting and clustering of drug responses, increasing discovery efficiency [25,26,27]. Additionally, deep learning approaches have been successfully applied to analyse complex social interactions in rodents using modifications in the DLC model [28].

In this study, we also modified the traditional OKR setup by introducing IR backlighting to enhance signal quality. IR illumination, which zebrafish cannot detect, enables bright-field imaging of larvae without interfering with the visual stimulus, with significant improvement of their eye tracking. This minimises background noise and improves contrast for image acquisition—an approach consistent with recent best practices in zebrafish behavioural assays [20].

Our approach, utilising CRISPR technology for rapid assessment in zebrafish larvae up to the age of 5dpf, offers a significant advantage in aligning with the 3R principles of animal research—Replacement, Reduction, and Refinement—that underpin UK research policy. By restricting experiments to early larval stages, we avoided the need for long-term maintenance of adult mutant lines, thereby reducing animal usage, housing time, and resource consumption. This strategy promotes more ethical and sustainable research practices, particularly in high-throughput screening contexts.

## 5. Conclusions

Altogether, our findings represent a significant advancement in zebrafish behavioural phenotyping. The deep learning pipeline we present is precise, scalable, and accessible, offering a powerful tool for studies involving pigmentation-deficient models, complex behavioural assays, and high-throughput phenotypic screening. The integration of IR illumination further optimises the signal-to-noise ratio in OKR recordings, ensuring reliable and reproducible results across laboratories. This work not only enhances zebrafish-based research in ocular and neurological disease models but also contributes to the standardisation of visual behaviour assays in the era of AI-enabled biology.

## Data Availability

The original contributions presented in this study are included in the article/Supplementary Material. Further inquiries can be directed to the corresponding author.

## Supplementary Materials

The following supporting information can be downloaded at: https://www.mdpi.com/article/10.3390/biology14060663/s1, Table S1: CRISPR guide information; Table S2: Stimulus paramters for IR vs NoIR set-up; Table S3: Stimulus paramters for comparing DLC, Stytra and OKRtrack; Table S4: Comparative Summary of Visual Tracking Methods.
