# Supplementary material for: A Deep Learning Approach to Measure Visual Function in Zebrafish
Source: Biology (Basel). 2025 Jun 9;14(6):663. doi: 10.3390/biology14060663 (PMC12189860; doi:10.3390/biology14060663)
Supplement: Supplementary file 1 [file biology-14-00663-s001.zip › Supplementary Table S4.pdf]

Supplementary Table S4: Comparative Summary of Visual Tracking Methods

Comparative overview of major tracking tools used in behavioural and visual phenotyping studies. This table highlights the strengths and limitations of several popular deep learning and image-based tracking methods, including object detectors (YOLO), markerless pose estimators (DLC, DeepPoseKit, DANNCE), and zebrafish-specific tools (Stytra, OKRtrack). Our selection of DeepLabCut was based on its high sub-pixel precision, ability to track pigment-deficient zebrafish, and flexible integration with varied experimental setups.

Method	Type of Tracking	Key Strengths	Limitations
YOLO	Object detection using bounding boxes	High-speed inference; accurate detection of multiple large objects	Not designed for keypoint tracking; low accuracy for small features such as eyes
OKRtrack	Contrast-based segmentation and ellipse fitting	Quick processing on standard CPUs; requires no training dataset	Highly dependent on image contrast; sensitive to lighting variation; not suitable for hypopigmented or low-contrast images
DeepLabCut	Markerless 2D keypoint-based pose estimation	Sub-pixel accuracy; user-defined keypoints; adaptable to lighting/pigmentation; leverages pre-trained networks	Requires manual annotation for training; computationally intensive; performance dependent on video quality and training set
Stytra	Real-time contrast-based object segmentation	Integrated stimulus-delivery and tracking; open source; accessible	Contrast-based; prone to failure in hypopigmented models; less precise for small

			anatomical features
DANNCE	3D pose estimation using multi-view deep networks	Accurate 3D trajectory estimation; handles occlusion; suitable for full-body tracking	Requires multi-camera setup; complex to implement; large data and compute overhead; less suitable for 2D eye tracking
DeepPoseKit	Markerless 2D pose estimation via keypoints	Designed for anatomical tracking in neuroscience/ethology; open source; customisable	Less widely adopted than DLC; requires labelled datasets; training is computationally intensive
